# Bayesian Finite Element Model Updating and Assessment of Cable-Stayed Bridges Using Wireless Sensor Data

**DOI:** 10.3390/s18093057

**Published:** 2018-09-12

**Authors:** Parisa Asadollahi, Yong Huang, Jian Li

**Affiliations:** 1 Department of Civil, Environmental, and Architectural Engineering, The University of Kansas, Lawrence, KS 66049, USA; p014a939@ku.edu; 2 Key Lab of Structural Dynamic Behavior and Control of the Ministry of Education, School of Civil Engineering, Harbin Institute of Technology, Harbin 150090, China; 3 Key Lab of Smart Prevention and Mitigation for Civil Engineering Disasters of the Ministry of Industry and Information, Harbin Institute of Technology, Harbin 150090, China

**Keywords:** Bayesian model updating, Bayesian model class assessment, Transitional Markov Chain Monte Carlo, cable-stayed bridge, prediction-error precision, structural health monitoring, wireless sensor network

## Abstract

We focus on a Bayesian inference framework for finite element (FE) model updating of a long-span cable-stayed bridge using long-term monitoring data collected from a wireless sensor network (WSN). A robust Bayesian inference method is proposed which marginalizes the prediction-error precisions and applies Transitional Markov Chain Monte Carlo (TMCMC) algorithm. The proposed marginalizing error precision is compared with other two treatments of prediction-error precisions, including the constant error precisions and updating error precisions through theoretical analysis and numerical investigation based on a bridge FE model. TMCMC is employed to draw samples from the posterior probability density function (PDF) of the structural model parameters and the uncertain prediction-error precision parameters if required. It is found that the proposed Bayesian inference method with prediction-error precisions marginalized as “nuisance” parameters produces an FE model with more accurate posterior uncertainty quantification and robust modal property prediction. When applying the identified modal parameters from acceleration data collected during a one-year period from the large-scale WSN on the bridge, we choose two candidate model classes using different parameter grouping based on the clustering results from a sensitivity analysis and apply Bayes’ Theorem at the model class level. By implementing the TMCMC sampler, both the posterior distributions of the structural model parameters and the plausibility of the two model classes are characterized given the real data. Computation of the posterior probabilities over the candidate model classes provides a procedure for Bayesian model class assessment, where the computation automatically implements Bayesian Ockham razor that trades off between data-fitting and model complexity, which penalizes model classes that “over-fit” the data. The results of FE model updating and assessment based on the real data using the proposed method show that the updated FE model can successfully predict modal properties of the structural system with high accuracy.

## 1. Introduction

Long-span bridges are important civil infrastructure systems as they are vital links in transportation networks. Bridge structures age due to excessive loading or environmental degradation; understanding their safety and serviceability performance is therefore necessary to achieve sustainable management and maintenance. As a result, high-fidelity structural Finite Element (FE) models of bridges are often critical to effective management of civil infrastructure systems by capturing their dynamic responses for conditions evaluation. However, modeling errors always exist in FE models, even though they are developed based on the best available knowledge from the design drawings and documents. The source of these errors could be simplification and idealization of connections and boundary conditions, and uncertainties in section geometries and material properties. While there are structural assessment methods developed to accommodate certain uncertainties [1,2], it is still critical to reduce modeling errors through FE model updating by calibrating uncertain structural model parameters based on measured data, such that the updated FE model produces reliable structural response predictions compared to the real structure. 

Despite a long history, development of algorithms for FE model updating continues to be an active area of research in structural engineering and structural dynamics. FE model updating is typically formulated as optimization problems with the goal of minimizing the discrepancies between measured properties from a real structure and those of the FE model. For example, Zhang et al. [3] updated the FE model of a cable-stayed bridge in Hong Kong using an improved sensitivity-based updating algorithm. Zapico et al. [4] investigated the selection of uncertain parameters on the model updating results of an experimental small-scale bridge based on measured time histories. Jang and Smyth [5] updated the full-scale FE model of a long-span bridge using a nonlinear inequality constraint equation to improve the consistency of the FE model with the real measured data. Perera et al. [6] proposed an FE updating method based on the combined use of static and dynamic responses. However, most of these methods belong to the conventional deterministic methods that consider only a point estimate of the FE model parameters.

For real-world applications of FE model updating for large-scale bridges, an inherent challenge is that there are always modeling uncertainties involved, meaning that the exact behavior of a structure cannot be modeled using the incomplete information available. As a result, it is always necessary to take these uncertainties into account in model updating applications. There are various possible sources for these model uncertainties such as simplifying assumptions in construction of structural FE models; limited number of sensors for data collection from the structure; measurement noise; and changes in physical parameters such as mass and stiffness due to environmental and operational variations. Subsequently, researchers have been motivated to tackle the challenge from a Bayesian perspective (e.g., [7,8,9,10,11]). In Bayesian FE model updating, Bayes’ Theorem is employed to consider all plausible values of the uncertain parameters and compute their Bayesian posterior probability density function (PDF), where the measured structural response data and initial knowledge about the uncertain structural model parameters are utilized to calibrate the FE models. Compared with deterministic FE model updating methods, Bayesian FE model updating tracks all plausible values of the FE model parameters based on the measured data and also explicitly incorporates the uncertain prediction errors, as well as measurement errors, enabling more robust predictions of structural responses. 

For exploring Bayesian model updating in bridge structures, Behmanesh and Moaveni [12] performed probabilistic damage identification based on Bayesian FE model updating on a footbridge by using different subsets of measured data. Jang and Smyth [13] employed Bayesian model updating using modal properties identified from four sets of measured data with nine sensors to improve a full-scale model of a suspension bridge. To the best of the authors’ knowledge, the two aforementioned studies are probably the only two applications of Bayesian FE model updating to full-scale bridges, though there are some other investigations by applying Bayesian framework for system identification and damage detection of full-scale bridges. For examples, Arangio and Bontempi [14] employed and leaned Bayesian neural networks for the identification of damage of a cable-stayed bridge. Kuok and Yuen [15] and Ni et al. [16] investigated modal identification and dynamic characteristics of cable-stayed bridges by Bayesian method. The limited real-world applications of Bayesian model updating on bridges is presumably due to the inherent challenges, i.e., proper treatment of posterior uncertainties of the prediction-error parameters and selection of an appropriate class of structural models.

In Bayesian model updating and assessment, it is important to explicitly quantify the uncertain prediction-errors which are the difference between the response of the real system (measured data) and the predicted response of the analytical model, since no model of the structural system is expected to give perfect predictions of structural responses. The prediction-error precision parameters have been shown to play an important role for the effectiveness of Bayesian model updating [11,17]. To date, researchers have investigated three treatments of prediction-error precisions in the context of Bayesian FE model updating. The first treatment is to assign or estimate constant values to prediction-error precisions [18]. The second one is to consider prediction-error precisions as additional uncertain parameters and update them together with the structural model parameters in a Bayesian inference framework [10,12,18]. The most advanced one is to analytically marginalize over prediction-error precisions and update the corresponding hyper-parameters to account for the full posterior uncertainty of the prediction-error precision parameters in an effective manner [19]. However, it is based on the conjugate probability distributions and an analytical solution for the posterior PDF was derived. In this study, we investigate the case that it is intractable to get an analytical posterior PDF when marginalizing over the prediction error precision parameters. In this case, for characterizing the posterior PDFs of model parameters, Transitional Markov Chain Monte Carlo (TMCMC) sampler is applied. In addition, this research compares the above-mentioned three treatments for prediction-error precisions explicitly based on the bridge structural FE model and uses the new treatment of prediction-error parameters for real-world Bayesian FE model updating for a full-scale cable-stayed bridge. 

Effective applications of model updating are also highly dependent on the selection of the class of structural models. Structural model classes are a set of predictive input–output probability models for a structural system based on different parameterization of a structure. As an example, Jang and Smyth [13] utilized a sensitivity-based cluster analysis to group 249 mass and stiffness related parameters in order to reduce the number of uncertain parameters for model updating of a suspension bridge. On the other hand, Bayesian model class selection technique has been developed to quantify the plausibility of different model classes based on the measured data [20,21]. An information-theoretic interpretation [8] shows that the posterior probability of each model class depends on the difference between a measure of the average data-fit of the model class and the amount of information extracted from the data by the model class, which penalizes model classes that “over-fit” the data. In other words, loosely speaking, models should be no more complex than is sufficient to explain the data. This is important to alleviate the effects of measurement noise and environmental effects in the real data. However, there are still limited studies which have focused on model class selection explicitly in bridge engineering. In this work, together with a sensitivity-based cluster analysis, Bayesian model class assessment is performed. We choose a set of candidate model classes and calculate their posterior probability based on the data by applying Bayes’ Theorem at the model class level. 

This paper performs Bayesian model updating and assessment on a full-scale FE model for a long-span cable-stayed bridge based on both synthetic and real structural health monitoring (SHM) data. In Section 2, we present a framework for Bayesian FE model updating and assessment and a robust Bayesian inference method is proposed which marginalizes the prediction-error precisions and uses TMCMC sampler for characterization of the posterior PDFs of the structural model parameters. Two other treatments of prediction-error precision parameters are also introduced and compared with the proposed method from a theoretical point of view. In Section 3, Bayesian FE model updating performance is compared by using each of the three prediction-error precision treatments based on synthetic data and TMCMC sampler. Two model classes are defined with different parameter groupings through clustering the results of sensitivity analysis. Finally, Bayesian FE model updating based on a set of long-term recorded data from a dense wireless sensor network (WSN) on the long-span cable-stayed bridge is performed given each of the two candidate model classes. TMCMC is utilized to draw posterior samples of the structural model parameters and estimate the evidence value for each model class. Model class assessment is then performed to quantify the relative plausibility of the two competing model classes and to choose the more plausible model class. Concluding remarks are made in Section 4.

## 2. Bayesian Framework for FE Model Updating and Assessment

### 2.1. Structural Model Class 

We employ low amplitude vibration testing and suppose that $N_{s}$ sets of measured vibration time histories are available from a structure and $N_{m}$ modes of vibration are identified from each vibration time histories so that we have modal data $Y_{r}$ for each data segment $\left(r=1,\ldots ,N_{s}\right),$ which includes a vector of natural frequencies ${\hat{\omega }}_{r}^{2}={\left[{\hat{\omega }}_{r,1}^{2},\ldots ,{\hat{\omega }}_{r,N_{m}}^{2}\right]}^{T}\in {\mathbb{R}}^{N_{m}\times 1}$ and a vector of mode shapes: ${\hat{\varphi }}_{r}={\left[{\hat{\varphi }}_{r,1}^{T},\ldots ,{\hat{\varphi }}_{r,N_{m}}^{T}\right]}^{T}\in {\mathbb{R}}^{N_{m}N_{o}\times 1}$, where ${\hat{\varphi }}_{r,i}\in {\mathbb{R}}^{N_{o}}$ gives the identified mode shape components of the $i^{th}$ mode ($i=1,\ldots ,N_{m}$) at the $N_{o}$ measured DOFs (degrees of freedom).

As part of the definition of the structural model class M, we choose a set of linear structural models with classical normal modes, which is a good approximation because we use small-amplitude vibrations recorded from the structural system. Each model has an unknown global mass matrix $M\in {\mathbb{R}}^{N_{d}\times N_{d}}$ and stiffness matrix $K\in {\mathbb{R}}^{N_{d}\times N_{d}}$ parameterized by a set of uncertain structural model parameters θ, which include both mass-related parameters, i.e., deck mass, and stiffness-related parameters, i.e., Young’s modulus and moments of inertia in the global lateral and vertical directions. Based on the defined global stiffness and mass matrices, the eigenvalue equation for the ith mode is given by:

(1) $$\;K\left(\theta \right)\varphi_{i}=\omega_{i}^{2}M\left(\theta \right)\varphi_{i},\;\;\;\;\;\;i=1,\ldots ,N_{m}\;$$

which governs the deterministic relation between modal parameters $\omega_{i}^{2}$ and $\varphi_{i}\in {\mathbb{R}}^{N_{d}\times 1}$ with the structural model parameter vector θ. A probability model can be chosen for the prediction of modal parameter from θ by selecting a PDF for the prediction-errors that maximizes Shannon’s entropy subject to some prior constraints, i.e., using stochastic embedding of the parameterized deterministic model [8]. It is seen that each modeling of parameter vector θ (e.g., a sub-structuring) corresponds to a set of stochastic predictive model $\left\{p\left(y|\theta ,M\right):\theta \in {\mathbb{R}}^{N_{d}}\right\},$ where **y** is the model prediction of the modal parameters. A stochastic model class M [8] for the structural system is therefore defined that consists of this set of predictive model $\left\{p\left(y|\theta ,M\right):\theta \in {\mathbb{R}}^{N_{d}}\right\}.$

### 2.2. Bayesian Modeling 

The initial probability information of the model parameter vector θ is expressed by the *prior distribution* p(θ). In this study, uniform PDFs are assigned as prior distributions for each component in θ. Since the stiffness-related parameters are typically modeled with higher confidence in FE models, narrower intervals are assigned for the corresponding uniform distributions. The *likelihood function*, $p\left(D_{N_{s}}|\theta ,\rho ,\eta \right),$ comes from substituting the measured modal data $D_{N_{s}}=\left\{Y_{1},\ldots ,Y_{N_{s}}\right\}$ into a stochastic model p(y|θ,ρ,η) for prediction y given specified model parameter vector θ. By employing the maximum entropy probability model [22] subject to the first two moment constraints, the uncertain prediction-errors for measured frequencies ${\hat{\omega }}_{r}^{2}$ and mode shapes ${\hat{\varphi }}_{r}$ are modeled as independent and identically distributed zero-mean Gaussian vector with unknown covariance matrices $\rho^{-1}I_{N_{m}}$ and $\eta^{-1}I_{N_{m}N_{o}},$ respectively, where ρ and η are prediction-error precision parameters for ${\hat{\omega }}_{r}^{2}$ and ${\hat{\varphi }}_{r}$, respectively. Note that equal prediction-error parameters are assumed for each mode and each data segment. Inspired by Vanik et al. [23], it is also assumed that the modal data is independently distributed from data set to data set, mode to mode and from frequency to mode shape when conditional on the structural model parameter vector θ. The likelihood function for θ based on $D_{N_{s}}$ is given by:

(2) $$\begin{array}{l} p\left(D_{N_{s}}|\theta ,\rho ,\eta \right)=\prod_{r=1}^{N_{s}}p\left(Y_{r}|\theta ,\rho ,\eta \right)\\ =\prod_{r=1}^{N_{s}}\left(p\left({\hat{\omega }}_{r}^{2}|\theta ,\rho \right)p\left({\hat{\varphi }}_{r}|\theta ,\eta \right)\right)\\ =\prod_{r=1}^{N_{s}}\left(N\left({\hat{\omega }}_{r}^{2}|\omega^{2}\left(\theta \right),\frac{1}{\rho }I_{N_{m}}\right)N\left({\hat{\varphi }}_{r}|\Gamma \varphi \left(\theta \right),\frac{1}{\eta }I_{N_{m}N_{o}}\right)\right)\; \end{array}$$

where $\omega^{2}\left(\theta \right)\in {\mathbb{R}}^{N_{m}\times 1}$ and $\varphi \left(\theta \right)\in {\mathbb{R}}^{N_{d}N_{m}\times 1}$ are analytical natural frequencies and mode shapes computed from the structural model parameters θ, respectively; $\Gamma \in {\mathbb{R}}^{N_{m}N_{0}\times N_{m}N_{d}}$ is the selection matrix formed from 1’s and 0’s to pick the measured DOFs from the full analytical mode shape φ(θ). This likelihood function in (2) measures how well the model for specified model parameters θ predicts the modal data $D_{N_{s}}$. The uncertainties in the prediction-error precision parameters ρ and η are modeled by the following Exponential hyper-priors:

(3) p(ρ)=Exp(ρ|τ)=τexp(−τρ) 

(4)  p(η)=Exp(η|ν)=νexp(−νη) 

which are the maximum entropy distributions with support [0,∞) for given mean values $\;\tau^{-1}$ and $\nu^{-1}$ of ρ and η, respectively.

### 2.3. Bayesian Updating and Model Class Assessment

The modal data $D_{N_{s}}$ can be used to update the relative plausibility of each structural model by computing the posterior PDF $p\left(\theta |D_{N_{s}},\rho ,\;\eta \right)$ using Bayes’ Theorem:

(5) $$p\left(\theta |D_{N_{s}},\rho ,\;\eta \right)=c^{-1}p\left(D_{N_{s}}|\theta ,\rho ,\;\eta \right)p\left(\theta \right)\;$$

where the prior PDF p(θ) and the likelihood function $p\left(D_{N_{s}}|\theta ,\rho ,\;\eta \right)$ for θ are defined in the previous subsection. In (5), c is a normalizing constant for the posterior PDF $p\left(\theta |D_{N_{s}},\rho ,\;\eta \right)$, which is equal to the *evidence* (or marginal likelihood) function $p\left(D_{N_{s}}|\rho ,\;\eta \right)$ and computed by the Total Probability Theorem:

(6) $$\;c=p(D_{N_{s}}|\rho ,\;\eta )=\int p\left(D_{N_{s}}|\theta ,\rho ,\;\eta \right)p\left(\theta \right)d\theta \;$$

The computation of the multi-dimensional integral in (6) is non-trivial. If there is no analytical solution for (5) and the data $D_{N_{s}}\;$ provides less independent information than needed to constrain the updated parameter vector θ [24], the stochastic simulation methods are practical to calculate the model class evidence, such as Transitional Markov Chain Monte Carlo (TMCMC) method [25,26].

The likelihood function in (2) for the modal data $D_{N_{s}}$ and the prior on θ define a *stochastic model class* M(ρ, η) for the structural model. However, there is always uncertainty in terms of which parameterized model class to choose to represent a structural system (e.g., different sub-structuring for obtaining θ and different values of ρ and η). One can also choose a set of candidate model classes $\left\{M_{m},\;m=1,\ldots ,M\right\}\;$ and calculate their posterior probability $p\left(M_{m}|D_{N_{s}}\right)\;$ to quantify their plausibility based on the data by applying Bayes’ Theorem at the model class level. This is known as Bayesian *model class assessment* or *model class selection*. If different model classes are assigned equal plausibility a priori, $p\left(M_{m}|D_{N_{s}}\right)\propto p\left(D_{N_{s}}|M_{m}\right)$ and then model class assessment is equivalent to comparing the evidence values $p\left(D_{N_{s}}|M_{m}\right)\;$ for candidate model classes, which implements Bayesian Ockham Razor. A recent interesting information-theoretic interpretation [8] shows that the logarithmic function of the evidence function $p\left(D_{N_{s}}|M_{m}\right)$ is expressed as follows:

(7) $$\;\ln p\left(D_{N_{s}}|M_{m}\right)=E[\ln p\left(D_{N_{s}}|\theta ,M_{m}\right)]-E[\ln\frac{p\left(\theta |D_{N_{s}},M_{m}\right)}{p(\theta |\;M_{m})}]\;$$

where the expectations E(·) are taken with respect to the posterior PDF $p\left(\theta |D_{N_{s}},M_{m}\right)$. The first term $E[\ln p\left(D_{N_{s}}|\theta ,M_{m}\right)]$ is the posterior mean of the log likelihood function, which is a measure of the average data-fit of the model class $M_{m}$, and the second term $E\left[\ln\frac{p\left(\theta |D_{N_{s}},M_{m}\right)}{p(\theta |\;M_{m})}\right]$ is the Kullback-Leibler information of the posterior relative to the prior, which measures the amount of information gain about θ from the data $\;D_{N_{s}}$ when performing model updating and is related to the model complexity. Therefore, the evidence explicitly builds in a trade-off between the data-fit of the model class and its information-theoretic complexity, which is important in model updating applications. If two model classes explain the measured data equally, the simpler model class is preferred, since the other model class consists of unnecessary model complexity. Presence of unnecessary model complexity generally leads to data over-fitting and the subsequent response predictions may then be unreliable due to excessive dependence on the details of the specific data (for model updating), e.g., measurement noise and environmental effects. On the other hand, over-simple model cannot fit the data $\;D_{N_{s}}$ well (data-fit measure is small) and the trade-off between data-fitting and model complexity may also not be the optimal one that maximizes the log evidence in (7). Therefore, Bayesian model class assessment has a built-in penalty against models that are too simple (“under-fit” the data) and too complex (“over-fit” the data). In this work, Bayesian model class assessment is performed to choose the most plausible model class among various competing ones, which are defined from different structural model parameter groupings, i.e., different dimensionality of the model parameter vector θ.

### 2.4. Proposed Bayesian Inference Method by Using a New Treatment of Prediction-Error Precision Parameters and TMCMC Sampler

It is known that no model of a structural system is expected to give perfect predictions because of complex dynamic behavior, e.g., the traffic loading induced vibration and the temperature effect. Therefore, in Bayesian FE model updating explicitly quantifying the uncertain prediction-errors is important, which is the difference between the real system output and the model output. Note that the uncertainty of the prediction-error parameter has a close relation with the posterior uncertainty quantification of the model parameters θ from Bayes’ Theorem in Equation (5). For example, less prediction accuracy due to heavy traffic loading and temperature effects will produce larger posterior uncertainties of θ, which indicate lower confidence in the FE model updating. Here we study the treatment for the uncertain prediction-error parameters.

In this paper, we investigate a new treatment that integrates out the prediction-error precisions (inverse variances) ρ and η in Equation (2) as “nuisance” parameters. The primary purpose is to consider all plausible prediction-error parameter values more efficiently rather than simply estimating them and hence achieve higher robustness in model updating. To get an analytical solution for the marginalization of the prediction-error precisions, the likelihood function in (2) is rewritten as:

(8) $$\begin{array}{c} \;p\left(D_{N_{s}}|\theta ,\rho ,\eta \right)\;=\prod_{r=1}^{N_{s}}\left(N\left({\hat{\omega }}_{r}^{2}|\omega^{2}\left(\theta \right),\frac{1}{\rho }I_{N_{m}}\right)N\left({\hat{\varphi }}_{r}|\Gamma \varphi \left(\theta \right),\frac{1}{\eta }I_{N_{m}N_{o}}\right)\right)\\ =N\left({\hat{\omega }}^{2}|T\omega^{2}\left(\theta \right),\frac{1}{\rho }I_{N_{m}N_{s}}\right)N\left(\hat{\varphi }|\Psi \varphi \left(\theta \right),\frac{1}{\eta }I_{N_{m}N_{o}N_{s}}\right)\; \end{array}$$

where ${\hat{\omega }}^{2}={\left[{\left({\hat{\omega }}_{1}^{2}\right)}^{T},\ldots ,{\left({\hat{\omega }}_{N_{s}}^{2}\right)}^{T}\right]}^{T}\in {\mathbb{R}}^{N_{m}N_{s}\times 1}$ and $\hat{\varphi }={\left[{\hat{\varphi }}_{1}^{T},\ldots ,{\hat{\varphi }}_{N_{s}}^{T}\right]}^{T}\in {\mathbb{R}}^{N_{m}N_{o}N_{s}\times 1};$ the selection matrix $\Psi ={\left[\Gamma^{T},\ldots ,\Gamma^{T}\right]}^{T}\in {\mathbb{R}}^{N_{m}N_{o}N_{s}\times N_{m}N_{d}}$; $Τ={\left[I_{N_{m},}\ldots ,I_{N_{m}}\right]}^{T}\in {\mathbb{R}}^{N_{m}N_{s}\times N_{m}}\;$ is the transformation matrix between the vector of $N_{s}$ sets of identified natural frequencies ${\hat{\omega }}^{2}$ and the analytical natural frequencies $\omega^{2}\left(\theta \right)$. By marginalizing over ρ and η, the likelihood function in (8) becomes:

(9) $$\begin{array}{c} p\left(D_{N_{s}}|\theta \right)\;=\int p\left({\hat{\omega }}^{2}|\theta ,\rho \right)p\left(\rho |\tau \right)d\rho \cdot \int p\left(\hat{\varphi }|\theta ,\eta \right)p\left(\eta |\nu \right)d\eta \;\\ \;=\int N\left({\hat{\omega }}^{2}|\omega^{2}\left(\theta \right),\frac{1}{\rho }I_{N_{m}N_{s}}\right)\mathrm{Exp}\left(\rho |\tau \right)d\rho \cdot \int N\left(\hat{\varphi }|\varphi \left(\theta \right),\frac{1}{\eta }I_{N_{m}N_{o}N_{s}}\right)\mathrm{Exp}\left(\eta |\nu \right)d\eta \\ \;=\mathrm{St}\left({\hat{\omega }}^{2}|\omega^{2}\left(\theta \right),\frac{1}{\tau }I_{N_{m}N_{s}},2\right).\mathrm{St}\left(\hat{\varphi }|\varphi \left(\theta \right),\frac{1}{\nu }I_{N_{m}N_{o}N_{s}},2\right)\; \end{array}$$

which results in the product of two Student’s t-distributions both with degrees of freedoms of 2 for natural frequencies and mode shapes, respectively. Note that theoretically it is better to have diagonal covariance matrices with different variances for various modes, which can provide more accurate modeling of the prediction errors. However, the unique prediction errors ρ and η are selected to produce analytical solutions of the marginalization in Equation (9). Based on the analytical likelihood function in Equation (9) and the prior PDF for θ, the structural model parameters can be updated by using Bayes’ theorem as:

(10) $$p\left(\theta |D_{N_{s}}\right)\propto p\left(D_{N_{s}}|\theta \right)p\left(\theta \right)\;$$

The posterior PDF $p\left(\theta |D_{N_{s}}\right)\;$ is intractable to get an analytical solution; however, it can be characterized by the samples obtained from Markov Chain Monte Carlo (MCMC) sampler [27], which is a class of stochastic simulation method. The marginal posterior of θ can also be expressed as:

(11) $$p\left(\theta |D_{N_{s}}\right)=\int p\left(\theta |D_{N_{s}},\rho ,\;\eta \right)p\left(\rho ,\;\eta |D_{N_{s}}\right)d\rho d\eta \;$$

It is seen that the contribution of posterior uncertainties of ρ and η from the posterior PDF $p\left(\rho ,\;\eta |D_{N_{s}}\right)\;$ is preserved properly by integrating them out, instead of seeking to estimate or sample these two “nuisance” parameters. Therefore, model updating with this treatment should have higher parameter estimation accuracy. Regarding posterior uncertainty quantification, the posterior uncertainty of the marginal posterior PDF $p\left(\theta |D_{N_{s}}\right)$ is always larger than that of the posterior $p\left(\theta |D_{N_{s}},\rho ,\eta \right),$ especially when the posterior uncertainty of ρ and η is large. In addition, the accurate characterization of uncertainties of ρ and η is easy to be achieved using this treatment since we don’t need to draw huge amount of samples for these two parameters when using MCMC algorithm. Therefore, this treatment of prediction-error precisions tends to provide accurate posterior uncertainty quantification for the structural model parameters θ and alleviates the over-confidence and under-confidence problems in the parameter estimation. The numerical results given later support these conclusions because the model updating results of this method outperforms others. 

MCMC has received much attention for Bayesian model updating in recent years, by which samples consistent with the posterior distribution of the model parameters are generated. An advantage of MCMC methods is that they can provide a full characterization of the posterior uncertainty, no matter the data available is sufficient to constraint the updated parameters [8], thereby they are practical for the cases where no analytical solutions are available for the posterior PDFs. Several MCMC methods have been proposed with the goal of improving the computational efficiency of posterior sampling in Bayesian model updating [25,26,28,29]. Among these methods, TMCMC [25] is motivated by a related adaptive Metropolis-Hastings method [28], which works for various types of PDFs such as very peaked, flat, and multimodal PDFs. The algorithm is applicable for problems with high dimensions and also enables model class assessment by providing an estimation of the multi-dimensional integral in (6) for the evidence value as a by-product. 

The fundamental basis of TMCMC is that samples are taken from a series of intermediate PDFs in an adaptive manner, expressed as:

(12) $$\;p_{j}\left(\theta |D_{N_{s}}\right)\propto p\left(\theta \right)p{\left(D_{N_{s}}|\theta \right)}^{s_{j}}\;j=0,\ldots ,J;\;0=s_{0}<s_{1}<\ldots <s_{J}=1\;$$

where *j* is the stage number and $s_{j}$ is the corresponding tempering parameter for the *j*th stage, which controls the speed of this gradual transition and is automatically computed in the process to form the intermediate PDFs. It can be seen from (12) that the series start with prior PDF p(w|U)  (when $j=0\;\mathrm{and}\;s_{0}=0)\;$ and converge to the posterior PDF $p\left(w|D_{N_{s}}\right)$ (when $j=J\;\mathrm{and}\;s_{J}=1$). Note that normalization is not necessary since the MCMC requires only the relative probability densities.

Given the *j*th stage samples $\left\{\theta_{j,1},\;\theta_{j,2},\;\ldots ,\;\theta_{j,N}\right\}$ from $p_{j}\left(\theta |D_{N_{s}}\right),$ the $p_{j+1}\left(\theta |D_{N_{s}}\right)\;$ samples of the (*j* + 1)th stage are obtained by a resampling approach, which can be achieved as follows [25]: the plausibility weights of samples $\left\{\theta_{j,1},\;\theta_{j,2},\;\ldots ,\;\theta_{j,N}\right\}$ with respect to $p_{j+1}\left(\theta |D_{N_{s}}\right)$ are first computed according to:

(13) $$\;v(\theta_{j,k})=\frac{p{\left(D_{N_{s}}|\theta_{j,k}\right)}^{s_{j+1}}p\left(\theta_{j,k}\right)}{p{\left(D_{N_{s}}|\theta_{j,k}\right)}^{s_{j}}p\left(\theta_{j,k}\right)}=p{\left(D_{N_{s}}|\theta_{j,k}\right)}^{s_{j+1}-s_{j}},\;k=1,\ldots ,\;N\;$$

Having the plausibility weights, the uncertain model parameters are then resampled based on the normalized weight such that:

(14) $$\theta_{j+1,k}=\theta_{j,l}\;\mathrm{with}\;\mathrm{probability}\;\;\frac{v(\theta_{j,l})}{\sum_{l=1}^{N}v(\theta_{j,l})},\;\;k=1,\ldots ,\;N\;$$

where *l* = dummy index. It can be seen that if N is large, samples $\left\{\theta_{j,1},\;\theta_{j,2},\;\ldots ,\;\theta_{j,N}\right\}$ will be generated according to the intermediate PDF $p_{j}\left(\theta |D_{N_{s}}\right)$.

The expectation of $v(\theta_{j,l})$ can be estimated by the average of $\theta_{j,k\;}$samples, k=1,…, N:

(15) $$E\left(v(\theta_{j,k})\right)=\frac{\int p{\left(D_{N_{s}}|\theta \right)}^{s_{j+1}}p\left(\theta \right)d\theta }{\int p{\left(D_{N_{s}}|\theta \right)}^{s_{j}}p\left(\theta \right)d\theta }\approx \frac{1}{N}\sum_{k=1}^{N}v(\theta_{j,k})\;$$

Finally, the evidence p(D|U) can be evaluated as:

(16) $$\begin{array}{c} \;p\left(D_{N_{s}}\right)=\frac{\int p\left(D_{N_{s}}|\theta \right)p\left(\theta \right)d\theta }{\int p\left(\theta \right)d\theta }=\frac{\int p{\left(D_{N_{s}}|\theta \right)}^{s_{J}}p\left(\theta \right)d\theta }{\int p{\left(D_{N_{s}}|\theta \right)}^{s_{0}}p\left(\theta \right)d\theta }\\ \;=\frac{\int p{\left(D_{N_{s}}|\theta \right)}^{s_{1}}p\left(\theta \right)d\theta }{\int p{\left(D_{N_{s}}|\theta \right)}^{s_{0}}p\left(\theta \right)d\theta }\frac{\int p{\left(D_{N_{s}}|\theta \right)}^{s_{2}}p\left(\theta \right)d\theta }{\int p{\left(D_{N_{s}}|\theta \right)}^{s_{1}}p\left(\theta \right)d\theta }\ldots \frac{\int p{\left(D_{N_{s}}|\theta \right)}^{s_{J}}p\left(\theta \right)d\theta }{\int p{\left(D_{N_{s}}|\theta \right)}^{s_{J-1}}p\left(\theta \right)d\theta }\\ =\prod_{j=1}^{J}E\left(v(\theta_{j,k})\right)\; \end{array}$$

More details about TMCMC sampler are referred to [25]. 

Note that the Bayesian FE model updating framework in this paper is based on the assumption that the structural model parameters are time-invariant. For the situation with changing structural properties, Kalman filter formulation may need to be employed which can track the changing state of the system and the estimates are updated using a state transition model and measurements. In this paper, we use the identified modal parameters from acceleration data collected during a one-year period from the large-scale WSN on the bridge, and it is assumed that the structural properties are invariant during the one-year period. In fact, the variation of the structural model parameters for real applications is one of important source of the model uncertainties which can induce larger posterior uncertainties of the structural model parameters θ and the FE model predictions, i.e., less confidence in the inference of θ and subsequent model predictions.

### 2.5. Two Other Treatments for the Uncertain Prediction-Error Precision Parameters

Two other treatments for the uncertain prediction-error parameters are also investigated in the paper. The first treatment is to assign constant values for prediction-error precisions ρ and η in the likelihood function $p\left(D_{N_{s}}|\theta ,\rho ,\eta \right)$ in (2). By assuming equal error precisions for each mode and each data segment, two constant values are required for natural frequencies and mode shapes, which can be estimated based on the measured modal data directly. Inspired by Vanik et al. [18], the prediction-error precisions ρ and η are computed as follows:

(17) $$\rho =\frac{N_{s}N_{m}}{\sum_{r=1}^{N_{s}}\sum_{i=1}^{N_{m}}{\hat{\omega }}_{r,i}^{2}}\;$$

(18) $$\eta =\frac{N_{s}N_{m}}{\sum_{r=1}^{N_{s}}\sum_{i=1}^{N_{m}}\frac{\left|\right|{\hat{\varphi }}_{r,i}-\sum_{r=1}^{N_{s}}{\hat{\varphi }}_{r,i}/N_{s}{\left|\right|}^{2}}{\left|\right|{\hat{\varphi }}_{r,i}{\left|\right|}^{2}}}\;$$

where ||.|| is the Euclidean vector norm, so $\left|\right|x^{2}\left|\right|=x^{T}x.$ The main drawback of this treatment is that it ignores the posterior uncertainties of ρ and η by estimating these two error precision parameters directly from data. 

Another treatment is to update prediction-error precisions as additional uncertain parameters by Bayesian inference. Accordingly, two uncertain parameters ρ and η corresponding to the prediction-error precisions of frequencies and mode shapes are added together with the structural model parameters θ in the updating process as shown in the following:

(19) $$p\left(\theta ,\rho ,\;\eta |D_{N_{s}}\right)\propto p\left(D_{N_{s}}|\theta ,\rho ,\;\eta \right)p\left(\theta \right)p\left(\rho \right)p\left(\eta \right)\;$$

Using the probability product rule, we can rewrite the full posterior as:

(20) $$p\left(\theta ,\rho ,\;\eta |D_{N_{s}}\right)=p\left(\theta |D_{N_{s}},\rho ,\;\eta \right)p\left(\rho ,\;\eta |D_{N_{s}}\right)\;$$

It is seen that the posterior uncertainties of prediction-error precisions ρ and η have been quantified in the full Bayesian updating procedure. This is useful as the posterior PDF of θ will not be conditional on any specified values of ρ and  η, which may not always be an “optimal” choice for effective model updating. In this case, we need to sample ${\left[\theta^{T},\rho ,\;\eta \right]}^{T}$ using TMCMC rather than only θ.  However, compared with the strategy that marginalizes over prediction-error precision parameters ρ and η  rather than sampling them directly in the previous subsection, lower efficiency of MCMC sampling tends to be produced by using this strategy since the structural model updating procedure is much more sensitive to the prediction-error parameters ρ and η than the associated hyper-parameters τ and ν. 

## 3. Illustrative Examples 

### 3.1. Jindo Bridge FE Model 

**Figure 1:**
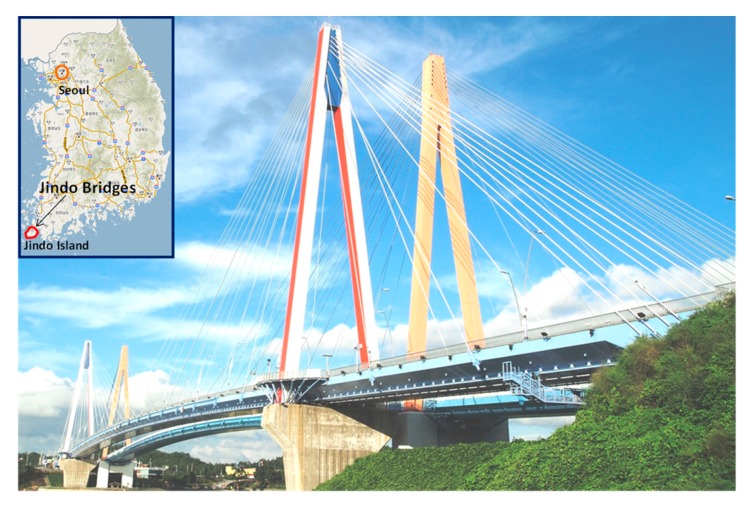
Jindo Bridges. The bridge on the left is the subject of this study.

Bayesian model updating is performed for the Jindo Bridge, which is a twin cable-stayed bridge in South Korea, as shown in Figure 1. The bridge on the left is investigated in this study because of the availability of the measured structural responses. The bridge has a 344-m main span and two 70-m side spans. 

**Figure 2:**
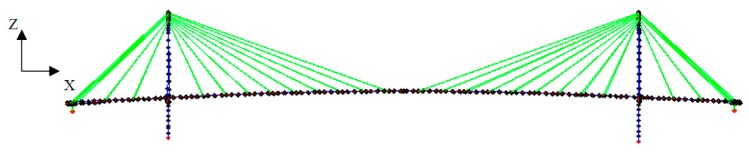
Finite element model of the Jindo Bridge.

The corresponding FE model of the bridge is demonstrated in Figure 2, which is constructed based on the available drawings and design documents using a MATLAB-based toolbox [30]. The toolbox was originally used to create the FE models for the benchmark control problems for seismic response of cable-stayed bridges [31,32]. The FE model consists of 641 nodes, 238 beam elements, 68 cable elements, 394 rigid links, and 641 lumped masses. In the model, deck-to-pylon connections are modeled using pin and roller supports. Analytical modal parameters including natural frequencies and mode shapes of the first 14 modes, including nine vertical, four lateral and one torsional vibration modes can be extracted from the bridge FE model as the simulated data for Bayesian FE model updating. These 14 modes are also the modes identified using response measurements from the field deployment, which will be described in Section 3.4.

### 3.2. Selection of Uncertain Structural Model Parameters

The selection of uncertain structural model parameters is part of the definition of the structural model class and plays an important role on effectiveness of the Bayesian FE model updating and accuracy of model for future response predictions. Therefore, a comprehensive sensitivity analysis is performed to determine the structural model parameters to be updated. To compute sensitivities, the parameter values are perturbed by ±10% and their corresponding natural frequencies are calculated through the FE model. Subsequently, the sensitivity of each parameter for each mode is obtained as the ratio between the frequency change and the parameter range. Moreover, complex structure systems such as long-span bridges are typically composed of a large number of structural members. Ideally, one would like to treat each structural member (e.g., girder beam elements) as an individual substructure in the FE model so that the physical properties (mass and stiffness) of each member can be inferred. However, the information from a limited number of sensors will generally be insufficient to support such a member-level resolution of parameter inference, so substructure assemblages, each consisting of multiple structural members may be necessary in order to reduce the number of model parameters in θ. Clustering analysis is therefore employed in this study to group the structural model parameters to form different model classes. Parameters with similar sensitivities are placed in the same cluster to ensure that the parameters from the same cluster have similar effects on the updating process [33]. 

Sensitivity analysis in conjunction with clustering is performed herein to generate a series of effective uncertain structural model parameters. Firstly, to perform sensitivity analysis, a set of physical parameters from the FE model is selected. The Jindo Bridge FE model contains 129 beam elements in the bridge girder. Since the bridge girder is symmetric with respect to the location of mid-span, both mass and stiffness (Young’s moduli and moments of inertia in local *x* and *y* directions) corresponding to the beam elements of half of the bridge girder are considered, leading to a total of 260 individual physical parameters. Subsequently, the sensitivities of these 260 parameters to the natural frequencies of the structure are analyzed.

**Figure 3:**
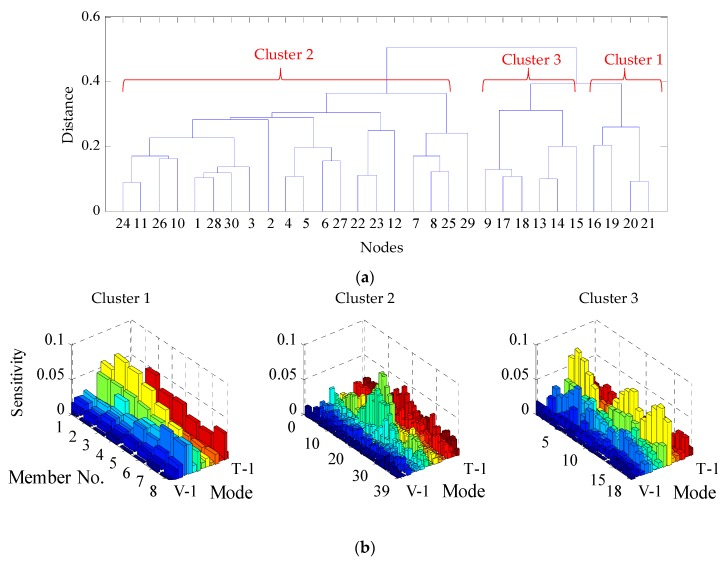
(**a**) Hierarchical binary tree for mass sensitivities; (**b**) Sensitivity matrices of mass clusters. The “Mode” axis indicates the first 9 vertical, 4 lateral and 1 torsional modes.

To perform clustering, cosine distance [34] is first used as a proximity measure to compute the closeness among the sensitivities. Then, hierarchical clustering is applied to determine the final clusters. Within the hierarchical clustering, the Un-weighted Pair Group Method with Arithmetic Mean (UPGMA) is employed to link the similar sensitivities. More specifically, the pairwise cosine distances of the sensitivities are calculated for each two parameters, by which the parameters are first grouped into a maximum of 30 binary clusters based on the similarity of sensitivities. In other words, if there are more than 30 parameters, some groups will contain more than one parameter. The horizontal axis of Figure 3a shows these 30 initial clusters. Subsequently, close binary clusters are put near each other in the horizontal axis of the binary tree and they are linked based on the UPGMA method to form the hierarchical tree. Distance criterion is used to form the final clusters. The distance threshold is selected as 0.4 for mass and 0.7 for all other physical parameters to produce a reasonable number of clusters.

**Figure 4:**
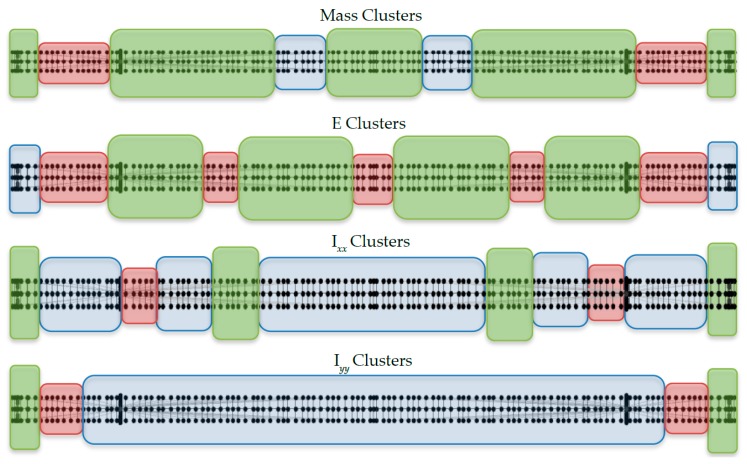
Cluster analysis result for four types of physical parameters for the bridge girder. Elements with the same color are from one cluster (blue box: cluster 1, green box: cluster 2, red box: cluster 3).

By performing separate clustering, three clusters for each type of physical parameters including mass, Young’s moduli, *I_xx_* (moment of inertia in x-direction), and *I_yy_* (moment of inertia in y-direction) and thereby 12 clusters in total are obtained. The binary tree to the three mass clusters is shown in Figure 3a. Figure 3b shows the sensitivity matrices of the three mass clusters. Note that clustering is based on the cosine distance of the sensitivity vectors with respect to 14 modes; the parameters within the same cluster show similar sensitivity to these modes on an average sense. As mentioned previously, individual mass parameters are assigned to the beam elements of half of the bridge girder, leading to 65 parameters for mass. In this figure, the x axis refers to these mass parameter numbers. The grouping results from the cluster analysis are presented in Figure 4 by showing the physical locations of each parameter group using different colors. 

Finally, two model classes $M_{1}$ and $M_{2}\;$ are defined based on the clustering results. Model class $M_{1}$ corresponds to a simplified class of structural models by selecting one parameter for each of the four types of physical parameters (mass, Young’s modulus, *I_xx_* and *I_yy_*). Thus, the number of uncertain parameter components in θ is only four in total. Regarding model class $M_{2}$, it consiss of 12 parameter components in θ corresponding to all the clusters shown in Figure 4. Table 1 tabulates the model parameter components for the bridge girder in model classes $M_{1}$ and $M_{2},$ respectively. As will be discussed later, in the Bayesian updating process, all uncertain structural model parameters are normalized to 10 using their initial physical values. The updated parameters will then be multiplied to their initial physical values to obtain the update model for further analysis.

### 3.3. Numerical Investigation on the Comparison of the Prediction-Error Precision Parameter Treatments

In this section, Bayesian FE model updating is performed on the bridge model. Three cases are considered corresponding to the three aforementioned treatments of prediction-error precisions, respectively, in which Case 3 considers the proposed method that marginalizes over the prediction error precision parameter. The likelihood function for the structural model parameters θ  is defined in (2) and (9) by using the synthetic modal data of 14 natural frequencies and mode shapes. Mode matching is employed to reorder the analytical modes based on the measured ones, in which Modal Assurance Criteria (MAC) is utilized. 

The simulated modal data is generated from model class $M_{2}$, since it has relatively higher complexity. To introduce modeling uncertainties, a coefficient is multiplied to the true value of each mass or stiffness parameter as shown in Table 2. The noisy modal data of the fourteen modes are simulated by adding 2% and 5% zero-mean Gaussian random noise to the analytical natural frequencies and mode shapes, respectively. By repeating this process, 10 sets of noisy modal data are generated. To perform Bayesian model updating, model class $M_{1}$ is employed and all uncertain structural model parameters are normalized to 10 using their nominal values. 

To characterize the posterior PDF of the structural model parameters, TMCMC sampler is employed for each case and 1000 samples are drawn in each stage. Uniform distributions are assigned to the prior for all normalized structural model parameter components in θ with a range of 8 to 12. For the second case with prediction-error precisions updated, the Exponential priors in (3) and (4) are assigned for ρ and η with hyper-parameters $\tau =\nu =10^{-10},$ that is, these priors are almost flat. While for the third case with error-precisions marginalized, the priors of hyper-parameters τ and ν  are taken as uniform over large open intervals that start at zero. The number of stages in TMCMC sampler corresponding to each case is approximately 20, meaning that in total 20,000 samples are required to be drawn for all stages. The overall computational cost of TMCMC for each case is about 15 h using a Dell computer equipped with an Intel^®^ Xeon^®^ CPU E3-1241 and 16 GB of RAM running a 64-bit version of Windows 7.

**Figure 5:**
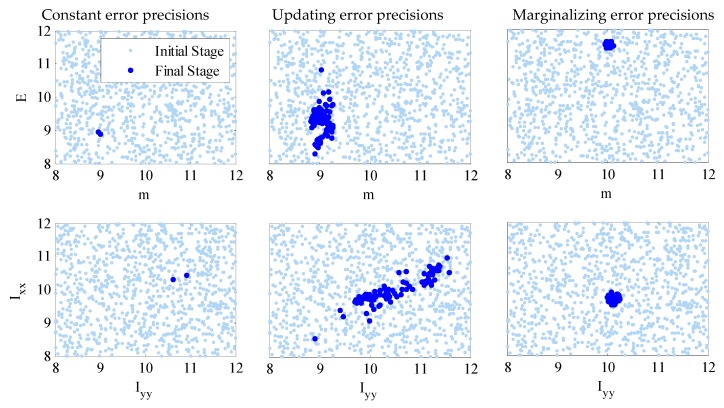
Samples of the initial and final stages of TMCMC for the three cases with constant, updating and marginalizing error precisions.

In Figure 5, the results of TMCMC samples of the structural model parameters in the first and last stages of TMCMC are demonstrated, which show how the samples converge from the prior to the posterior as the tempering parameter $s_{j}$ increases from 0 to 1. In this numerical investigation, about 20 stages were needed to achieve convergence. Compared with the case with error precisions updated, the samples are much more concentrated in the final stage for the case with the error precisions marginalized, indicating much higher identification confidence. The reason is presumably that the model parameters are much more sensitive to the prediction-error precisions ρ and η than their corresponding hyper-parameters τ and ν. This is consistent with our discussion in Section 2.4. However, for the case with constant precisions, the samples of I_yy_ versus I_xx_ at the last stage are even more concentrated, though at two incorrect locations. This is due to the fact that the uncertainties of the prediction-error precision parameters have been overlooked by estimating them directly, which leads to inaccuracy in the model updating results and underestimation of the posterior uncertainties of structural model parameters, as we discussed in Section 2.4. 

Table 3 tabulates the results in terms of the relative errors between the predicted natural frequencies by using the posterior mean of θ compared with the simulated natural frequencies, as well as the MAC values between the predicted and simulated mode shapes, for all three cases, where the error and MAC values for the *i*-th mode are computed as:

(21) $$e_{i}=\frac{\sum_{r=1}^{N_{s}}{\hat{\omega }}_{r,i}/N_{s}-\omega_{i}\left(\tilde{\theta }\right)}{\sum_{r=1}^{N_{s}}{\hat{\omega }}_{r,i}/N_{s}},\;i=1,\ldots ,N_{m}\;$$

(22) $${\mathrm{MAC}}_{i}=\frac{\;{\left({\left\{\sum_{r=1}^{N_{s}}{\hat{\varphi }}_{r,i}/N_{s}\right\}}^{T}\left\{\varphi_{i}\left(\tilde{\theta }\right)\right\}\right)}^{2}}{\left({\left\{\sum_{r=1}^{N_{s}}{\hat{\varphi }}_{r,i}/N_{s}\right\}}^{T}\{\sum_{r=1}^{N_{s}}{\hat{\varphi }}_{r,i}/N_{s}\}\right)\left({\left\{\varphi_{i}\left(\tilde{\theta }\right)\right\}}^{T}\left\{\varphi_{i}\left(\tilde{\theta }\right)\right\}\right)},\;i=1,\ldots ,N_{m}\;$$

where $\tilde{\theta }$ is the maximum a posteriori (MAP) value of the structural model parameter vector. It is seen that the MAC values are mostly larger than 0.9 for all cases, which are reasonably high. Regarding the frequency errors, the largest error (absolute value) is 6.91% and 6.70% for the cases of constant and updating error precisions, respectively. While for the advanced case with prediction-error precisions marginalized as “nuisance” parameters, the errors range from 0.3% to 3.6%, most of which are smaller than the corresponding values for the other two cases. 

From the comparison of the Bayesian updating results for the three cases, it is shown that the performance of the third case with prediction-error precision marginalized is superior. This is due to the fact that the posterior uncertainties of the error precision are preserved properly by integrating them as “nuisance” parameters. However, the first two cases with constant and updated error precisions have limitations that the results depend on the specified values of computed or sampled error-precisions. Therefore, prescribing a robust treatment for prediction-error precisions for Bayesian FE model updating is important because of the significant effect of the prediction error precision parameters, especially for large-scale complex civil structures such as long-span bridges. The merit of the new treatment with error precision marginalized is that the concern of the sensitivity of these parameters on the model updating performance has been alleviated significantly in a rigorous manner. 

### 3.4. Real-World Application of Bayesian FE Model Updating on Jindo Bridge

**Figure 6:**
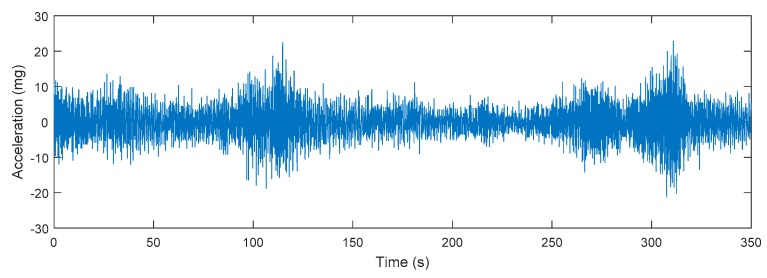
A sample measured acceleration time history from Jindo Deck on 5 June 2012.

In this section, Bayesian FE model updating is performed for Jindo Bridge by using long-term monitoring data from a dense deployment of a WSN that include 113 wireless sensor nodes, each composed of an Imote2 (MEMSIC) with an SHM-A [35] or SHM-H sensor board [36], both equipped with high-sensitivity accelerometers and with relatively higher sensitivity for the SHM-H. The sensor nodes were attached to the steel bridge surface using magnets. Data was collected during a twelve-month period starting from 1 September 2011 to 30 August 2012 is considered in this study. Figure 6 presents an example of the acceleration time histories from one channel measured on 5 June 2012 from the Jindo Deck. Details of the sensor network and data are presented in [37,38]. The Jindo Deck and Haenam Deck sensor networks were operated by two base stations and hence data collection was conducted independently between these two networks. Therefore, modal identification of Jindo Deck and Haenam Deck were performed separately. A combination of Natural Excitation Technique (NExT) [39] and Eigensystem Realization Algorithm (ERA) [40] was used to perform system identification of the bridge. Nine vertical, four lateral and one torsional modal properties of the deck were identified from the acceleration measurements. 

**Figure 7:**
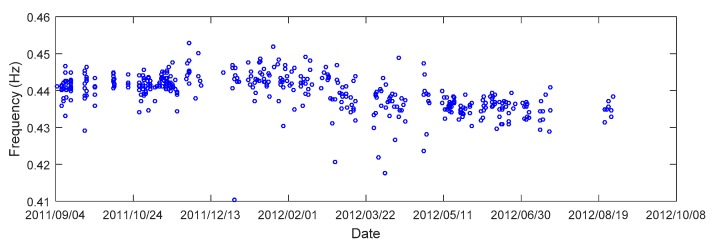
Identified modal frequencies of the 1st vertical mode during the monitoring period.

Environmental and operational conditions such as temperature and excitation amplitude have been recognized to potentially impact natural frequencies. The wireless smart sensor network measured the temperature during the deployment, offering an excellent opportunity to evaluate the relationship between modal frequencies and temperature. It was observed in Asadollahi and Li [38] that as temperature increased, the modal frequency decreased, while the excitation amplitude did not appear to have a clear relationship with the modal frequencies in this particular case. Therefore, the effect of temperature was removed from modal frequencies based on the linear relation observed between temperature and modal frequencies before performing Bayesian model updating. Complete descriptions of the modal identification results can be found in [38]. Figure 7 presents the identified modal frequencies from the 1st vertical mode during the one-year monitoring period as an example. The measured data for model updating typically consists of the extracted modal parameters including the natural frequencies and mode shapes since they significantly affect the dynamic response of the bridge structure. About 400 sets of modal data $\left(N_{s}=400\right)$ were identified from the long-term monitoring and are used in this study. 

Model updating problems are intrinsically ill-conditioned and often ill-posed when dealing with noisy measurement data, leading non-unique solutions. Under the Bayesian framework, incorporating as much prior knowledge of the structure as possible can help alleviate the ill-conditioning and ill-posedness [41,42]. Therefore, upper and lower bounds are defined for the prior uniform distributions of the structural model parameters. In particular, 5% variation from the nominal values (equal to 10) is considered for the stiffness related parameters because detailed and relative accurate information about the section and material properties of the deck and pylons is available. However, a larger range is selected for the bridge girder mass parameters since only mass of main structural members is considered in the initial FE model while other sources of masses are not. The unconsidered masses could come from structural elements such as deck wearing surface, wind fairings, cable anchors, stiffeners, splices and bolts, and non-structural elements including guard rails and barrier curbs, as well as the live loads (vehicular loads). Based on the length and width information of the deck, calculation indicates that the concrete deck slab alone could add approximately 40% of the considered mass from the structural elements. Therefore, we set the upper bound to 18, which means that we allow mass to be increased by up to 80% of the nominal value of element masses. The lower and upper bounds of the uniform prior for all model parameter components are tabulated in Table 4.

The modal data of all fourteen natural frequencies and the first three vertical mode shapes are used to construct the likelihood function $p\left(D_{N_{s}}|\theta \right)\;$in (9). The likelihood function is obtained by marginalizing the prediction-error precisions ρ and η since in the previous section this treatment was found to achieve the best Bayesian model updating performance. Two runs of TMCMC were performed to draw posterior samples (1000 samples per stage) for the structural model parameters θ corresponding to the two model classes $M_{1}$ and $M_{2}$. The number of stages for both runs were around 85, leading to a total of 85,000 samples drawn for all stages. 

**Figure 8:**
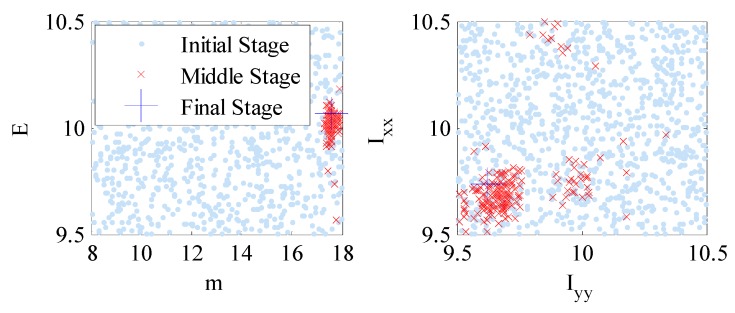
Plot of samples in the structural model parameter space generated at the initial, middle and final stages of TMCMC for model class $M_{1}.$

**Figure 9:**
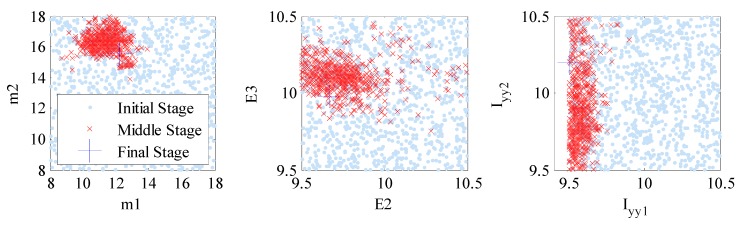
Plot of samples in the structural model parameter space generated at the initial, middle and final stages of TMCMC for model class $M_{2}.$

Figure 8 and Figure 9 show the samples of several structural model parameter components corresponding to the initial (stage number j=1), middle (stage number j≈0.5J, where J is the total number of stages) and final (stage number j=J) stages of TMCMC for the two runs corresponding to model classes $M_{1}$ and $M_{2},$ respectively. It can be seen that the region in the structural model parameter space with significant posterior probability content is very concentrated compared to the prior distribution of θ. This is achieved efficiently in TMCMC since it constructs a series of intermediate PDFs that gradually converge to the posterior PDF from the broad prior PDF. It is worthwhile noting that although the samples of each parameter component at the final stage of TMCMC look like only a single point as shown in Figure 6 and Figure 7, all samples are included in the figures. The concentration of the posterior samples is due to the availability of 400 sets of modal data in the model updating process, leading to high certainty in the posterior distributions. Compared with Figure 6 and Figure 7, the samples of the final stage in Figure 5 (numerical investigation) are more scattered since only 10 sets of modal data were employed in the updating process. In Table 5, we tabulate the posterior mean and coefficient of variation (C.O.V.) values of model parameter samples together with the percentage change of posterior means compared with those of the initial FE model for both model classes. It is observed that the C.O.V. values are generally smaller for model class $M_{2}$ than $M_{1}$, indicating smaller posterior uncertainties and higher confidence in the model updating results. In addition, the percentage change in structural model parameter components compared with the initial calibration values are relatively larger for model class $M_{2}$ (up to 79.54%), showing more information exacted from the available data.

By using the prior and posterior distributions of structural model parameters, prior and posterior predictions of modal parameters $D^{*}$ can be expressed in (23) and (24), respectively:

(23) $$\;p\left(D^{*}\right)=\int p\left(D^{*},\theta \right)d\theta =\int p\left(D^{*}|\theta \right)p\left(\theta \right)d\theta \;$$

(24) $$\;p\left(D^{*}|D_{N_{s}}\right)=p\left(D^{*},\theta |D_{N_{s}}\right)d\theta =\int p\left(D^{*}|\theta \right)p\left(\theta |D_{N_{s}}\right)d\theta \;$$

where $p\left(D^{*}|\theta \right)=\mathrm{St}\left({\left({\hat{\omega }}^{2}\right)}^{*}|\omega^{2}\left(\theta \right),\frac{1}{\tau }I,2\right).\mathrm{St}\left({\hat{\varphi }}^{*}|\varphi \left(\theta \right),\frac{1}{\nu }I,2\right)$ is the model predictive PDF which has the similar form of Equation (9); ${\left({\hat{\omega }}^{2}\right)}^{*}$ and ${\hat{\varphi }}^{*}$ are the predictive natural frequency and mode shape vectors, respectively. It is known from the sampling theory that the generation of samples $D^{*},$ which characterize the posterior prediction PDF $p\left(D^{*}|D_{N_{s}}\right)$ (prior prediction PDF $p\left(D^{*}\right)$), can be achieved simply by the following procedure: first draw samples of θ from the posterior PDF $p\left(\theta |D_{N_{s}}\right)$ (prior p(θ)), and then collect samples of $D^{*}$ generated from the predictive PDF $p\left(D^{*}|\theta \right)$, in which we incorporate the samples of θ sequentially. 

**Figure 10:**
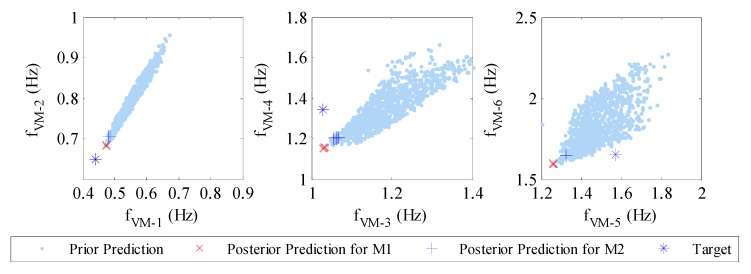
Prior and posterior predictions of natural frequencies for model class $M_{1}$ and $M_{2}.$

Figure 10 demonstrates the predicted values of natural frequencies of the first six vertical modes together with the target natural frequencies (identified from the data) for both model classes. Similar to θ samples, the predicted natural frequencies from the posterior samples are concentrated within relatively small regions, most of which are close to the target frequencies. By using the error index defined in (21), the errors of the prior and posterior prediction means of natural frequencies are computed and tabulated in Table 6 for both model classes. It is observed that the errors of posterior predictions are reduced significantly for most modes compared with the values of prior predictions. Only three modal frequency errors are not reduced in posterior predictions, which include the fifth vertical (VM-5) and fourth lateral (LM-4) modes as well as the torsional mode (TM-1). By comparing the results of the two model classes, the large absolute errors of the posterior predictions (e.g., modes VM-5, LM-3, LM-4 and TM-1) for model class $M_{2}$ are generally smaller than that of $M_{1}.$ In addition, the absolute errors of the posterior predictions for the lower modes are generally smaller than 10% for model class $M_{2},$ which is thought to be acceptable for damage assessment using these results. Meanwhile, it should be noted that the absolute errors in Table 6 are not as small as the values in the simulated data case (Table 3), this could be partially due to the higher noise content in the real-world SHM data for modal identification and model updating. A possible strategy for better model updating performance is to increase the quantity and quality of the available time-domain data and have more identified modes for model updating. 

In addition to the investigation of Bayesian model updating, Bayesian model class assessment is also studied to quantify the plausibility of proposed candidate model classes given the measured data. Based on the posterior samples of θ from TMCMC, the posterior probability $p\left(M_{m}|D_{N_{s}}\right),$ the log evidence $\ln p\left(D_{N_{s}}|M_{m}\right),$ the posterior mean of log likelihood $E[\ln p\left(D_{N_{s}}|\theta ,M_{m}\right)]$, and the relative entropy (information gain) $E\left[\ln\frac{p\left(\theta |D_{N_{s}},M_{m}\right)}{p(\theta |\;M_{m})}\right]$ are computed and compared in Table 7 for the two model classes $M_{1}$ and $M_{2}$. The primary purpose of this table is to compare the trade-off between the data-fit of the model class and the information-theoretic complexity for each model class to select a more optimal one. It is seen that model class $M_{2}$ produces a larger posterior mean of log likelihood and a larger relative entropy in comparison with those of $M_{1}$, indicating a better data fit and a higher model complexity for $M_{2}$. Regarding the trade-off between data fit and model complexity, the log evidence value of model class $M_{2}$ is much larger than that of $M_{1}$, implying that it gives a much better balance between the data-fit and the model complexity [8]. Although $M_{2}$ has a larger expected information gain (third row), showing that it extracts more information from the data than $M_{1}$, its mean data-fit is so much larger than that of $M_{2}$ (second row) because of more detail parameterization that its log evidence is dominant over that of $M_{1}$, making $M_{2}$ a much more plausible model class based on the data (first column). Since the posterior probability $p\left(M_{2}|D_{N_{s}}\right)$ is so much larger than $p\left(M_{1}|D_{N_{s}}\right),$ the model class $M_{2}\;$ which has more detailed parameterization is relatively more probable conditional on the data $D_{N_{s}}$ and so its contribution is dominant over that of $M_{1}$ when making response predictions of the bridge structure.

In summary, Bayesian FE model updating of the bridge using real-world data from long-term SHM leads to an accurate FE model which has vibration properties close to the real structure system and produces more reliable predictions of structural responses. In addition, the plausibility of competing model classes for the bridge system can also be assessed based on their posterior probability from Bayes’ Theorem, giving a rigorous method for structural model selection.

## 4. Conclusions

The presented work proposed a robust Bayesian inference method which marginalizes the prediction-error precisions and uses Transitional Markov Chain Monte Carlo (TMCMC) sampler. Bayesian FE model updating and assessment on a long-span cable-stayed bridge are performed by using both synthetic and real SHM data from a Wireless Sensor Network. This work is motivated from the fact that FE model calibration or updating for real civil structures is typically ill-conditioned and ill-posed when using noisy incomplete data because of various source of modeling uncertainties. Therefore, one should not just search for single “optimal” FE model parameters but rather attempt to describe the family of all plausible FE model parameters that are consistent with both the measured data and prior information. 

To achieve reliable Bayesian model updating performance for complex bridge structures, an advanced treatment of prediction-error precision parameters, named marginalizing error precisions was studied where the analytical solution of posterior probability density function (PDF) of model parameters is intractable. TMCMC was applied to draw samples from the posterior PDF of the structural model parameters. In addition, it is compared with two other existing treatments of prediction-error precisions, termed constant error precisions and updating error precisions, by investigating the associated Bayesian model updating performance. From both the theoretic analysis and simulation-based investigation, the treatment with prediction-error precisions marginalized has been found to produce the best model updating performance in terms of more accurate model parameter inference and posterior uncertainty quantification, hence achieving more reliable predictions of modal properties. 

In order to choose an appropriate class of models to describe the bridge system, Bayesian model class assessment has also been performed. Firstly, two model classes were defined from the parameter grouping results based on sensitivity-based clustering analysis. Bayesian FE model updating was then performed by marginalizing over prediction-error precisions given the two model classes using the identified modal data from long-term monitoring of the structure. A measure of plausibility for a class of models is given by the TMCMC sampler, which allows for rational comparisons of competing model classes based on the real data. The more probable model class gives a better balance between data-fit and model complexity. Overall, the results of FE model updating and assessment of the long-span bridge using real data measured from a long-term wireless monitoring system show that the updated FE model can successfully predict modal properties of the real structural system with high accuracy and reliability, by comparing them with the identified modal properties of the structure. 

## Figures and Tables

**Table 1:** Uncertain components in the structural model parameter vectors for model class $M_{1}$ and $M_{2}.$

Model Class	Bridge Girder Parameters											
	Mass			Young’s Modulus			Moment of Inertia					
$M_{1}$	*m*			*E*			*I_yy_*			*I_xx_*		
$M_{2}$	*m*_1_	*m*_2_	*m*_3_	*E*_1_	*E*_2_	*E*_3_	*I_yy_*_1_	*I_yy_*_2_	*I_yy_*_3_	*I_xx_*_1_	*I_xx_*_2_	*I_xx_*_3_

**Table 2:** Disturbance coefficients for structural model parameter components to introduce modeling uncertainties in model class $M_{2}.$

Uncertain Parameters in $M_{2}$		Coefficient
Mass	*m*_1_	1.1
	*m*_2_	1
	*m*_3_	0.9
Young Modulus	*E*_1_	1.05
	*E*_2_	1
	*E*_3_	0.95
Moment of Inertia	*I_yy_*_1_	0.95
	*I_yy_*_2_	1
	*I_yy_*_3_	1.05
	*I_xx_*_1_	1.05
	*I_xx_*_2_	1
	*I_xx_*_3_	0.95

**Table 3:** Frequency errors and MAC values using the three error precisions.

Mode *	Case with Constant Error Precisions		Case with Error Precisions Updated		Case with Error Precisions Marginalized	
	Freq. Errors (%)	MAC	Freq. Errors (%)	MAC	Freq. Errors (%)	MAC
VM-1	−6.91	0.997	−6.70	0.997	−0.30	1.000
VM-2	−2.60	0.997	−2.19	0.997	1.18	1.000
VM-3	−2.84	0.990	−2.53	0.992	0.31	0.999
VM-4	−1.51	0.993	−1.27	0.996	1.02	0.999
VM-5	−1.33	0.996	−0.85	0.996	0.87	1.000
VM-6	−1.08	0.994	−0.63	0.996	−0.45	0.950
VM-7	0.05	0.997	0.57	0.997	−0.75	0.994
VM-8	0.18	0.996	0.75	0.996	−2.46	0.990
VM-9	1.60	0.997	2.25	0.998	−2.00	0.974
LM-1	−6.54	0.998	−6.17	0.998	−3.56	1.000
LM-2	−3.87	0.998	−3.52	0.997	0.19	0.999
LM-3	−5.37	0.988	−4.99	0.988	0.27	0.999
LM-4	−4.75	0.985	−4.43	0.987	1.92	0.878
TM-1	−6.27	0.992	−5.93	0.992	1.42	0.987

* VM: Vertical Mode; TM: Torsional Mode; LM: Lateral Mode.

**Table 4:** Prior distribution bounds for the components in the structural model parameter vector θ.

Uncertain Parameters	$M_{1}$	$M_{2}$	Lower Bound	Upper Bound
Mass	*m*	*m*_1_	8	18
		*m*_2_		
		*m*_3_		
Young Modulus	*E*	*E*_1_	9.5	10.5
		*E*_2_		
		*E*_3_		
Moment of Inertia	*I_yy_*	*I_yy_*_1_	9.5	10.5
		*I_yy_*_2_		
		*I_yy_*_3_		
	*I_xx_*	*I_xx_*_1_	9.5	10.5
		*I_xx_*_2_		
		*I_xx_*_3_		

**Table 5:** Posterior Mean, C.O.V., and percentage change in structural model parameters.

Model Class $M_{1}$				Model Class $M_{2}$			
Parameters	Mean	C.O.V.	Changes (%)	Parameters	Mean	C.O.V.	Changes (%)
*m*	17.55	2.51 × 10^−3^	75.53	*m*_1_	12.20	8.42 × 10^−4^	21.97
				*m*_2_	15.55	4.51 × 10^−4^	55.55
				*m*_3_	17.95	3.97 × 10^−4^	79.54
*E*	10.06	1.92 × 10^−3^	0.65	*E*_1_	10.21	2.89 × 10^−4^	2.13
				*E*_2_	9.67	4.78 × 10^−5^	−3.32
				*E*_3_	9.98	5.01 × 10^−4^	−0.22
*Iyy*	9.62	8.03 × 10^−4^	−3.81	*Iyy*_1_	9.50	3.51 × 10^−4^	−4.96
				*Iyy*_2_	10.19	1.91 × 10^−4^	1.92
				*Iyy*_3_	9.67	2.56 × 10^−3^	−3.29
*Ixx*	9.74	1.46 × 10^−3^	−2.65	*Ixx*_1_	9.79	4.56 × 10^−4^	−2.14
				*Ixx*_2_	9.95	8.63 × 10^−4^	−0.46
				*Ixx*_3_	9.95	2.31 × 10^−4^	−0.46

**Table 6:** Identified frequencies, prior and posterior prediction means of frequencies, and the corresponding errors compared with the identified frequencies.

Mode	Identified Natural Freq. (Hz)	Prediction Means of Natural Frequencies (Hz) (Error %)		
		Prior Predictions	Posterior Predictions ($M_{1})$	Posterior Predictions ($M_{2})$
VM-1	0.44	0.61 (−39.12%)	0.47 (−7.76%)	0.48 (−9.87%)
VM-2	0.65	0.88 (−36.08%)	0.68 (−5.23%)	0.71 (−8.58%)
VM-3	1.03	1.35 (−31.10%)	1.03 (−0.18%)	1.06 (−2.63%)
VM-4	1.34	1.53 (−13.85%)	1.16 (13.71%)	1.20 (10.26%)
VM-5	1.57	1.67 (−6.45%)	1.26 (19.70%)	1.32 (15.75%)
VM-6	1.65	2.06 (−24.88%)	1.59 (3.46%)	1.65 (0.25%)
VM-7	1.88	2.59 (−37.77%)	1.93 (−2.88%)	2.00 (−6.15%)
VM-8	2.27	3.22 (−41.76%)	2.42 (−6.44%)	2.50 (−10.07%)
VM-9	2.82	3.72 (−31.88%)	2.78 (−1.27%)	2.81 (−0.22%)
LM-1	0.33	0.46 (−39.51%)	0.35 (−6.32%)	0.36 (−8.34%)
LM-2	0.82	1.17 (−42.81%)	0.89 (−8.70%)	0.88 (−7.18%)
LM-3	1.81	2.07 (−13.34%)	1.60 (−12.50%)	1.66 (−9.25%)
LM-4	3.36	2.95 (−12.33%)	2.24 (−33.34%)	2.32 (−31.09%)
TM-1	1.83	1.92 (−6.02%)	1.47 (−18.52%)	1.53 (−15.45%)

**Table 7:** Comparison of Bayesian updating results for model class $M_{1}$ and $M_{2}.$

Model Class	$\mathrm{Model}\;\mathrm{Class}\;\mathrm{Posterior}\;\mathrm{Probability}\;p\left(M_{m}|D_{N_{s}}\right)\;$	$\mathrm{Log}\;\mathrm{Evidence}\;E\left[\ln p\left(D_{N_{s}}|M_{m}\right)\right]$	$\mathrm{Data}-\mathrm{Fit}\;\mathrm{Measure}\;E\left[\ln p\left(D_{N_{s}}|\theta ,M_{m}\right)\right]$	$\mathrm{Information}\;\mathrm{Gain}\;E[\ln\frac{p\left(\theta |D_{N_{s}},M_{j}\right)}{p(\theta |\;M_{j})}]$
$M_{1}$	0.00	−394,469	−394,591	121
$M_{2}$	1.00	−357,125	−357,278	153
